# Influence of Music on Anxiety Induced by Fear of Heights in Virtual Reality

**DOI:** 10.3389/fpsyg.2015.01969

**Published:** 2016-01-05

**Authors:** Sofia Seinfeld, Ilias Bergstrom, Ausias Pomes, Jorge Arroyo-Palacios, Francisco Vico, Mel Slater, Maria V. Sanchez-Vives

**Affiliations:** ^1^Department of Systems Neuroscience, Institut d’Investigacions Biomèdiques August Pi i SunyerBarcelona, Spain; ^2^Event Lab, Department of Personality, Evaluation and Psychological Treatment, University of BarcelonaBarcelona, Spain; ^3^Department of Computer Science, University of MalagaMalaga, Spain; ^4^Institució Catalana de Recerca i Estudis AvançatsBarcelona, Spain; ^5^Department of Basic Psychology, Universidad de BarcelonaBarcelona, Spain

**Keywords:** music, anxiety, fear of heights, virtual reality, therapy

## Abstract

Music is a potent mood regulator that can induce relaxation and reduce anxiety in different situations. While several studies demonstrate that certain types of music have a subjective anxiolytic effect, the reported results from physiological responses are less conclusive. Virtual reality allows us to study diverse scenarios of real life under strict experimental control while preserving high ecological validity. We aimed to study the modulating effect of music on the anxiety responses triggered by an immersive virtual reality scenario designed to induce fear of heights. Subjects experienced a virtual scenario depicting an exterior elevator platform ascending and descending the total height of its 350 meters tall supporting structure. Participants were allocated to either a group that experienced the elevator ride with background music or without, in a between-groups design. Furthermore, each group included participants with different degrees of fear of heights, ranging from low to high fear. Recordings of heart rate, galvanic skin response, body balance, and head movements were obtained during the experiments. Subjective anxiety was measured by means of three questionnaires. The scenario produced significant changes in subjective and physiological measures, confirming its efficacy as a stressor. A significant increase in state anxiety was found between pre and post-assessment in the silence group, but not in the music group, indicating that post-stress recovery was faster in the musical group. Results suggest that music can ameliorate the subjective anxiety produced by fear of heights.

## Introduction

Anxiety is an adaptive and complex emotion consisting of physiological, cognitive, and behavioral components (for a review see, Spielberger and Reheiser, 2009). It is mainly characterized by feelings of tension, apprehension, nervousness and worry about potential negative outcomes or events (Spielberger et al., 1983). Anxiety sometimes accompanies stress responses that are triggered when the homeostasis of organisms is threatened by the presence of physical or psychological stressors. The main function of the stress system consists in re-establishing homeostasis by means of the autonomic nervous system and hypothalamic-pituitary-adrenal axis responses (Dickerson and Kemeny, 2004). Inappropriate basal activity and hyper-responsiveness of the stress system has been linked to anxiety disorders (Chrousos, 2009). Furthermore, it has also been shown that the exposure to acute stress can potentiate the anxiety responses due to the existence of overlapping structures and interactions between the stress and anxiety neurocircuitry (Grillon et al., 2007). Stress and anxiety are normal temporary reactions to potentially harmful situations. However, prolonged anxiety and stress reactions can have severe consequences for health, i.e. distortion of human homeostatic levels that might lead to both physical and mental impairments (for reviews see Shekhar et al., 2005; Katon et al., 2007; Lupien et al., 2009).

Various experimental studies have revealed that listening to music results in subjective, behavioral, and physiological changes related to stress and anxiety reduction (Zentner et al., 2008; Yehuda, 2011). In fact, music listening has been used as a tool for relaxation and anxiety management in a wide range of settings, e.g., hospitals, dentist clinics, and work offices (Nilsson, 2008). However, variations in experimental setups and types of music used in such studies render the interpretation of their results problematic. In his meta-analytic review, Pelletier (2004) concluded that music alone could significantly decrease arousal due to stress. However this effect depends on interactions with factors such as age, type of stress, combination with music assisted relaxation techniques, musical preference, previous musical experience, and type of intervention.

An early study attempting to measure the efficacy of music in modulating physiological arousal under a stressful situation was conducted by Thayer and Levenson (1983). In their study, the effects of two musical pieces on increasing or decreasing the degree of stress caused by watching a video were tested. Participants’ skin conductance responses (SCR) were found to increase when horror music was played and decrease with documentary music, compared to a silent condition. Their results suggest that music can be an effective means to modulate the perceived stress associated with watching a video. In line with this, Eifert et al. (1988) investigated the impact of positively appraised music in evaluative conditioning, i.e., how we can come to like something that we dislike, through an association. The study was conducted with people who have animal phobia, revealing that preferred music included in an *in vivo* exposure session could invoke a positive affective response that increased the efficacy of the treatment. A possible mechanism to explain these outcomes could be that when music comes into a direct conflict with negative stressful conditions, the arousal might be shifted (Yamamoto et al., 2007).

Furthermore, listening to self-selected relaxing music and classical music immediately after a brief cognitive stressor (mental challenging test) resulted in an increased feelings of relaxation and a reduction in state anxiety, when compared to groups who sat in silence or listened to heavy metal music (Labbé et al., 2007). The results obtained by Burns et al. (2002) were along the same lines; a significant effect caused by silence, classical music, and self-selected music was found in the state anxiety of subjects after exposure to a cognitive stressor in comparison to hard rock. Nonetheless, no significant changes were found in the physiological responses between groups in these studies, which suggest that music only had an influence on a subjective level.

However, a series of studies have demonstrated that music can also prevent significant increases in physiological responses due to anxiety. Knight and Rickard (2001) exposed subjects to a cognitive stressor (preparation for an oral presentation) in the presence of classical music or silence. The music condition was found to prevent significant increases in state anxiety, heart rate (HR), systolic blood pressure, and salivary immunoglobulin A. Similar results were obtained by Lai et al. (2008) where they studied the effect of lento music on reducing examination anxiety during a test. A within-subject design, where all participants did an exam under a silence and a music condition, was used. Their results indicate that subjective anxiety and pulse rates were lower with music, suggesting that it prevented stress and anxiety reactions produced by an exam situation. Nevertheless, in this study there was a possible placebo effect since subjects were previously informed about music’s potential benefits.

Additionally, other studies have demonstrated that music can facilitate recovery from a psychologically stressful task by modulating the post-stress responses of the hypothalamic pituitary adrenal axis and cardiovascular system. Researchers found significant decreases in salivary cortisol and systolic blood pressure in subjects who recovered from a laboratory stressor with relaxing background music in comparison to silence (Khalfa et al., 2003; Chafin et al., 2004). Along these lines, it has also been demonstrated that listening to relaxing classical music prior to a standardized stressor can affect the autonomic nervous system (Thoma et al., 2013). Finally, Allen and Blascovich (1994) carried out a within subjects study in which they evaluated surgeons’ responses to a serial subtraction task in the presence of silence, preferred music or experimenter selected music. Their results indicate that the playing of participants’ preferred music, and also experimenter selected music, reduced cardiovascular reactivity during the stressful task and improved performance.

One of the major problems in psychological research is that of trying to link ecological validity to experimental control (Campbell et al., 1963). The majority of studies carried out to observe the effects of music under a stressful situation have been done under laboratory conditions with artificially induced anxiety, often lacking external and ecological validity, or in natural settings where control over experimental conditions is difficult (Pelletier, 2004). Virtual reality (VR) systems allow us to recreate different circumstances of real life, where people respond in a realistic manner, and at the same time it allows us to study them under strict experimental conditions (Botella et al., 1998; Rovira et al., 2009; Bohil et al., 2011; Slater et al., 2014).

Immersive virtual reality (IVR) can evoke the sense of presence, which refers to behaving and feeling as if we were in a virtual world created by computer displays (for review see: Sanchez-Vives and Slater, 2005; Slater et al., 2009). An application that illustrates this phenomenon is the use of virtual reality exposure therapy (VRET) in which a therapist can expose patients to a virtual environment depicting a situation that triggers their anxiety response and, at the same time, maintain a high degree of control over the exposure sessions (Powers and Emmelkamp, 2008). There is now considerable scientific evidence that shows that VRET can be as effective as *in vivo* exposure therapy in the treatments of acrophobia, fear of flying, or post-traumatic stress disorder (Krijn et al., 2004; Rizzo et al., 2009; Meyerbröker and Emmelkamp, 2010). Additionally, VR has the advantage of allowing researchers to recreate situations that in real life would be impossible or dangerous (Botella et al., 1998; Slater et al., 2006).

The present study was aimed at comparing the effects on self-reported anxiety, physiological reactions and behavioral measurements, in an immersive virtual reality scenario depicting a height situation that either included background relaxing music or did not. Previous studies have shown the existence of significant differences in anxiety measures during immersive virtual reality experiences depicting elevators in subjects with different levels of fear of height (Wilhelm et al., 2005). Since this type of situation is not experienced as being stressful for everybody, in our study we aimed to also control for the differential effects that music might have depending on the variation in pre-existing fear of heights. For these reasons we selected relaxing music since it has been suggested that this plays an important role in reducing anxiety, through promoting positive emotions and parasympathetic activity (Knight and Rickard, 2001).

Our hypothesis was that listening to relaxing music during and after an experience of going up to a great height on an open elevator at the side of a building should prevent significant increases in anxiety and sympathetic nervous system arousal, compared to experiencing the situation without music.

## Materials and Methods

### Participants

Participants were recruited over email or from around the University campus. Before arriving at the experiment, participants were asked to complete the anxiety subscale of the Acrophobia Questionnaire (AQ; Cohen, 1977). The main inclusion criterion for this study was that participants were aged ≥ 18 years, and that they had an Acrophobia Questionnaire score between 0 and 60. Rates over 60 in AQ were excluded to avoid the presence of gravely acrophobic individuals in the study (Menzies and Parker, 2001; Jackson, 2009). Furthermore, other exclusion criteria for this study were the intake of psychotropic drugs, a history of epilepsy to avoid potential side-effects of virtual reality, or the intake of more than one unit of alcohol 6 h before participation in the study.

A total of 129 individuals answered the AQ, from which 46 subjects were finally selected based on their scores, fulfillment of inclusion and exclusion criteria, and their availability. Six persons had to be eliminated from the study due to technical problems with the VR system, leaving a final sample of 40 subjects. The experiment was a one-factor with two levels between-groups design. The two experimental conditions of the design were whether subjects were exposed to the scenario without music or in the presence of background relaxing music. Twenty participants were allocated quasi-randomly to one of the two experimental conditions based on their level of fear of heights measured by the AQ score. This was done in order to ensure that both groups were balanced, and included subjects with various degrees of fear of heights. **Table 1** summarizes the demographic information of the participants across the two conditions. This study followed ethical standards and was approved by the Ethical Committee for Clinical Research of Hospital Clinic of Barcelona, and participants gave informed written consent.

**Table 1:** The following table shows the participant’s distribution by gender among the groups as well as the mean (*M*) and standard deviations (*SD*) for Age, and Acrophobia Questionnaire (AQ) scores.

Group	Gender	Age	AQ
Music	12 females; 8 males	*M* = 27.15; *SD* = 3.03	*M* = 29.15; *SD* = 15.34
No-music	15 females; 5 males	*M* = 25.40; *SD* = 4.20	*M* = 29.35; *SD* = 16.37

### Materials

The experiment was conducted in the Event Lab Cave-like projection system (Cruz-Neira et al., 1992). This is a multi-person, high-resolution, virtual reality environment consisting of a cuboid-room of 3.80 m × 2.25 m × 3.80 m. The walls are rear-projection screens and the floor a down-projection screen in which Christie Digital Mirage WU3 three-chip digital light processing projectors throw full-color images with a resolution of 1920 × 1200 pixels, giving a 1.85 mm pixel size to the surrounding composite image. As a viewer wearing a tracking sensor moves within its display boundaries, the correct perspective and stereo projections of the environment are updated, and the image moves with and surrounds the viewer. Head tracking was performed with an Intersense IS 900 and a pair of CrystalEyes4 by Real3D stereo shutter glasses were used to separate the alternate fields going to the eyes. Physiological data was acquired using a NeXus-4 (MindMedia BV, Herten, Netherlands) physiological device with two electrodes in the palmar areas of the index and ring fingers of the right hand to record electrodermal actitivity (EDA). Electrocardiogram (ECG) was obtained with a g.MOBIlab device (g.tec—Guger Technologies OEG, Graz, Austria) and a bipolar ECG recording with an abdominal placement set up: the positive electrode was placed below the ribs on the left, the ground electrode at the same level on the right, and the negative electrode was placed on the upper sternum area. A custom Physio Recording Matlab Simulink Model (described in Spanlang et al., 2014) was used to process and record the physiological signals at a sample rate of 256 Hz. Oﬄine analysis of the physiological signals were carried out using the gBSanalyze software from g.tec and customized using MATLAB (Mathworks, Inc., Natick, MA, USA) scripts. Finally, with the aim of taking balance measurements, we used a Wii Balance Board (Nintendo) as a force platform.

**FIGURE 1:**
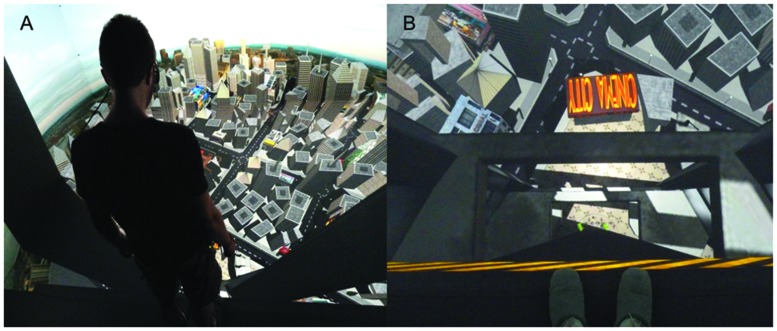
**(A)** View of the city from the top floor. The participant is wearing the active-stereo glasses for stereoscopic vision. For the sake of clarity, the image has been displayed in mono; **(B)** First person perspective of the elevator ascending. Notice that the feet of the participant can be seen at the border of the virtual elevator, blending in with the virtual environment.

The virtual environment used for this experiment depicted an exterior elevator platform ascending and descending the total height of its supporting structure -350 m tall (**Figures 1A,B**). The building had a total of nine floors. To add realism to the experience we included a background noise of an elevator moving and stopping through each floor, in both conditions. In order to take baseline measures we also created a neutral virtual setting on the ground floor that depicted a terrace with tables and chairs.

Musical stimuli for this experiment were obtained from the Infinitunes repository, powered by Melomics technology (Diaz-Jerez, 2011). Music was parameterized based on past studies in order to have a relaxing effect on the listener, e.g., it had a slow tempo around 50 bpm, it was only instrumental, it was predominantly composed of low tones, it did not have percussion and was presented at a mild volume of 60 db (Knight and Rickard, 2001; Nilsson, 2008; Elliott et al., 2011). A full description of the musical piece can be found in **Table 2**. These themes are particularly interesting for music studies, since they are new musical compositions that should not elicit any previous emotional meaning due to being totally unfamiliar to the participants. The piece of music used was validated in a previous study where most of the participants judged it as relaxing (Bergstrom et al., 2014).

**Table 2:** Summary of the specific musical parameters used to develop a relaxing and sedative musical piece.

Musical parameters	Analysis
Structure	A + A′ + A″ + A′″ where A: abcb′c′. A progressive structure were no identical repetition exists but it always uses the same material
Register	Re3-Si6
Complexity	Low technical complexity
Phrases	Irregular, absence of question, and answer
Loudness	Medium
Accent	4/4
Tempo	50 bpm
Tones duration	1/4 1/8 1/16
Tonality	M
Harmony	No conclusive harmonies and absence of tonal resolutions
Instrumentation	Celesta, harp, strings, choirs
Genre	Lullaby
Texture	Counterpoint. The melodies of violins and choruses are harmonized by chords of first inversion

### Measures

#### State Trait Anxiety Inventory-Form Y (STAI-Y)

The State Trait Anxiety Inventory-Form Y (STAI-Y) is a self-report survey containing two independent 20-item scales that measure state and trait anxiety. This inventory was developed by Spielberger et al. (1983) and has been adapted for the Spanish population with good test-retest reliability (Cronbach alpha of 0.90 for the state anxiety scale and 0.84 for the trait anxiety scale) and validity (Seisdedos, 1988). Scores around 20 indicate absence of anxiety, while scores closer to 80 represent high levels of anxiety. In the present study, subjects completed the state anxiety scale before (*preSTAI*) and after the VR exposure (*postSTAI*) to assess possible differences between conditions in the post-stress recovery response. The measure of interest, is the difference between the post-experiment total score and the pre-experiment total score (*dstai* = *Poststai*-*Prestai*). The more negative the values, the less anxiety experienced after the virtual scenario. Participants also answered the trait anxiety subscale before going through the VR experience.

#### Subjective Units of Discomfort (SUDS)

Wolpe (1958) developed the SUDS (subjective units of discomfort) as a method to measure changes related to anxiety levels during the application of systematic desensitization. Currently, SUDS are extensively used as a measure of emotional discomfort against specific stimuli (Tanner, 2012). The SUDS scores are obtained by asking the subject to read an 11 points scale ranging from 0 to 100, where 0 represents a state of absolute relaxation and 100 denotes a state of the worst anxiety experienced. This scale has shown acceptable validity and reliability in previous studies showing significant relationships with the Multiple Affect Adjective Check List (MAACL), the STAI (Kaplan, 1995) and the Minnesota Multiphasic Personality Inventory (Tanner, 2012). Subjects had to assess their level of anxiety with the SUDS scale at each floor.

#### Autonomic Perception Questionnaire (APQ)

The Autonomic Perception Questionnaire (APQ) is a 24-item visual analog scale used for the assessment of self-awareness of physiological activation (heart rate, perspiration, temperature change, respiration, gastrointestinal, muscle tension, and blood pressure). High scores in APQ indicate greater awareness of bodily sensations and correlates positively with anxiety, heart rate and skin conductance response (Mandler et al., 1958). In the study, subjects had to complete this questionnaire before and after going through the VR experience to assess how the stressor modified their levels of physiological self-awareness. The measure of interest is the difference between the post-experiment total score and the pre-experiment total score (*dAPQ* = *PostAPQ*-*PreAPQ*). The more negative the values, the less physiological self-awareness during the VR experience.

#### Virtual Reality Experience Questionnaire

With the objective of taking into account the degree of presence that participants experienced during this study, we included a 7 point Likert-type questionnaire that subjects had to fill in at the end of the session. **Table 3** summarizes the specific items included in this questionnaire. This type of information is useful in VR research since it provides further data to understand how people react in virtual environments. A similar strategy was used in Slater et al. (2009) and in Pan et al. (2012). Finally, only in the music group we also asked for the level of attention paid to the musical stimulus (7 point Likert-type scale, ranging from did not pay attention to I paid attention all the time), degree of liking of the musical piece (7 point Likert-type scale, ranging from nothing to very much) and the extent to which they judge the music as being relaxing (7 point Likert-type scale, ranging from relaxing to stressful).

**Table 3:** Summary of the specific questions included in the VR questionnaire that participants filled in after the virtual heights experience.

Variable	Questions		
Presence	1. During the VR experience, I had a stronger sense of being in…		
	The real world of the laboratory	1 2 3 4 5 6 7	The virtual world
Realism	2. During the VR experience the elevator felt real to me…		
	At no time	1 2 3 4 5 6 7	Almost all the time
Stressfulness	3. The feeling of ascending/descending in the virtual elevator seemed to me…		
	Not at all stressful	1 2 3 4 5 6 7	Extremely stressful
Fear of falling	4. To what extent did looking down at the edge of the platform feel the same as it would have in a similar situation in real life…		
	Not at all stressful	1 2 3 4 5 6 7	Extremely stressful
Real vs Virtual	5. The sense of fear of falling I experienced was…		
	Non-existent	1 2 3 4 5 6 7	Very high
Attention to scene	6. Please state how much attention did you paid to the VR scenario during the experience...		
	Not paying attention at all	1 2 3 4 5 6 7	Paying all my attention
Attention to sound	7. Please state how much attention did you paid to the sound of the elevator during the experience...		
	Not paying attention at all	1 2 3 4 5 6 7	Paying all my attention
Attention to beep	8. Please state how much attention did you paid to the beep sound during the experience...		
	Not paying attention at all	1 2 3 4 5 6 7	Paying all my attention

#### Physiological Responses

The physiological measures recorded in this experiment were electrodermal activity (EDA) and electrocardiogram (ECG). EDA responses are widely used as indirect measures of emotional arousal through changes in skin conductance, since they are strongly related to sympathetic responses. In this study, we measured and analyzed skin conductance level (SCL) which is related to a person’s overall arousal (Schmidt and Walach, 2000).

From the ECG signal the main measurement analyzed was heart rate, defined as beats per minute (bpm). Past studies have shown that HR is a reliable measure of stress in response to a threating situation in virtual reality and real environments (Meehan et al., 2002; Taelman et al., 2009). To carry out ECG analysis we automatically detected the QRS (ventricular contraction) complexes in the ECG time series based on a modified Pan-Tompkins algorithm (Pan and Tompkins, 1985). Subsequently, a visual inspection of the QRS detected was carried out, to correct missing or wrongly assigned points. Based on this method we determined the distance in time from one heart contraction to the next one (RR intervals).

The physiological recordings were over each of the first 30 s after the subject arrived at each floor. Also during this time he had to judge his or her level of anxiety while looking down. In total there were 20 trials per subject, including: baseline, each individual floor, which was visited twice (while ascending and descending), and a last trial taken on the ground floor. Furthermore, to eliminate within group variability in arousability, we analyzed the percentage of change from baseline in HR and SCL rather than absolute values. Normalization was carried out based on the literature using the following formula: Percentage of change = 100 × [(absolute value - baseline)/baseline] (Wiederhold et al., 2002; Davidson et al., 2003).

#### Standing Balance Parameters

With the aim of measuring behavioral parameters related to fear of heights we used a force platform (see above) to register center of pressure (CoP) coordinates. It has been demonstrated that significant changes occur in CoP displacements or postural stability as a result of anxiety and fear induced by standing on the edge of an elevated surface and also that individuals suffering from acrophobia might have prior abnormalities in balance control (Bles and Kapteyn, 1980; Boffino et al., 2009). Furthermore, Cleworth et al. (2012) demonstrated that virtual environments that simulated height situations can elicit very similar changes in postural control as real environments. Based on past studies, we decided to use two measurements of postural stability and sway: root mean square of medial-lateral sway and root mean square of anterior-posterior sway (Simeonov et al., 2005).

As with physiological measures, the analysis was done over 30 s windows, with a total of 20 trials per subject, and to control for within-group variability, normalization of the data was carried out following the same formula as above.

### Procedure

Each participant was asked to sit down in a quiet room and read a brief explanation of the experiment. They were requested to sign a consent form if they were willing to participate. In the brief explanation, participants were told that they could withdraw from the experiment at any time without explanation. Upon agreement, we first administered the STAI and then the APQ. Participants were also instructed in how to report their Subjective Units of Discomfort (SUDS). After participants had finished answering the pre-questionnaires they put on the physiological devices, shutter glasses and a microphone to communicate with the experimenter. Subsequently, participants entered the Cave system. They were instructed to stand on the Wii Board with the toes of their feet protruding from the edge (see **Figure 1B**). A 2-min baseline measurement was taken in a neutral virtual scenario, located on the ground floor. Afterward, the actual experiment started with the elevation of the virtual platform to the top of the building, stopping at all floors on the way. The elevator then followed the reverse path to the ground floor. Subjects remained for an average of 40 s on each floor during which a beep sound prompted them to state out loud their SUDS score. Finally subjects were asked to again complete the post-experiment questionnaires of the STAI and APQ. They also completed a questionnaire that gave basic demographic data such as age, gender, nationality, computer literacy, programming literacy, medication intake, alcohol intake, and presence or absence of any medical condition. Finally, they filled in a questionnaire to assess their perception of the virtual experience.

### Statistical Analysis

Due to the ordinal nature of the responses of the virtual reality experience questionnaire, we analyzed each questionnaire item using ordered logistic regression which included the factor Group (music and no-music) and the Acrophobia Questionnaire (AQ) as a covariate in order to control for participants’ different degrees of fear of heights. Ordered logistic regression is the appropriate method for the analysis of ordinal scales from questionnaire data since the assumptions of the standard general linear model (regression and ANOVA) that the responses be on an interval scale and normally distributed are obviously violated.

The Subjective Units of Discomfort, Heart Rate, Skin Conductance, and Balance Parameters, were analyzed using a mixed effects model with fixed effect Group (music and no music) and Direction (ascending and descending). The random effects were defined within individuals. In this analysis, we included the “Floor Level” as a covariate. The floor level could potentially influence the degree of anxiety since participants tend to report increased levels of anxiety on higher floors.

Finally, for analyzing the State Trait Anxiety Inventory and Autonomic Perception Questionnaire responses, a one-way ANCOVA was carried out. In this analysis, AQ was also included as a covariate in order to control for the degree of fear of heights.

One participant from the music condition was excluded from the analysis, since she was an outlier in some questionnaire responses and we realized that she was not a native Spanish speaker. This left a total sample of *n* = 19 in the music condition and *n* = 20 in the no music condition.

## Results

### Virtual Reality Experience Questionnaire

**FIGURE 2:**
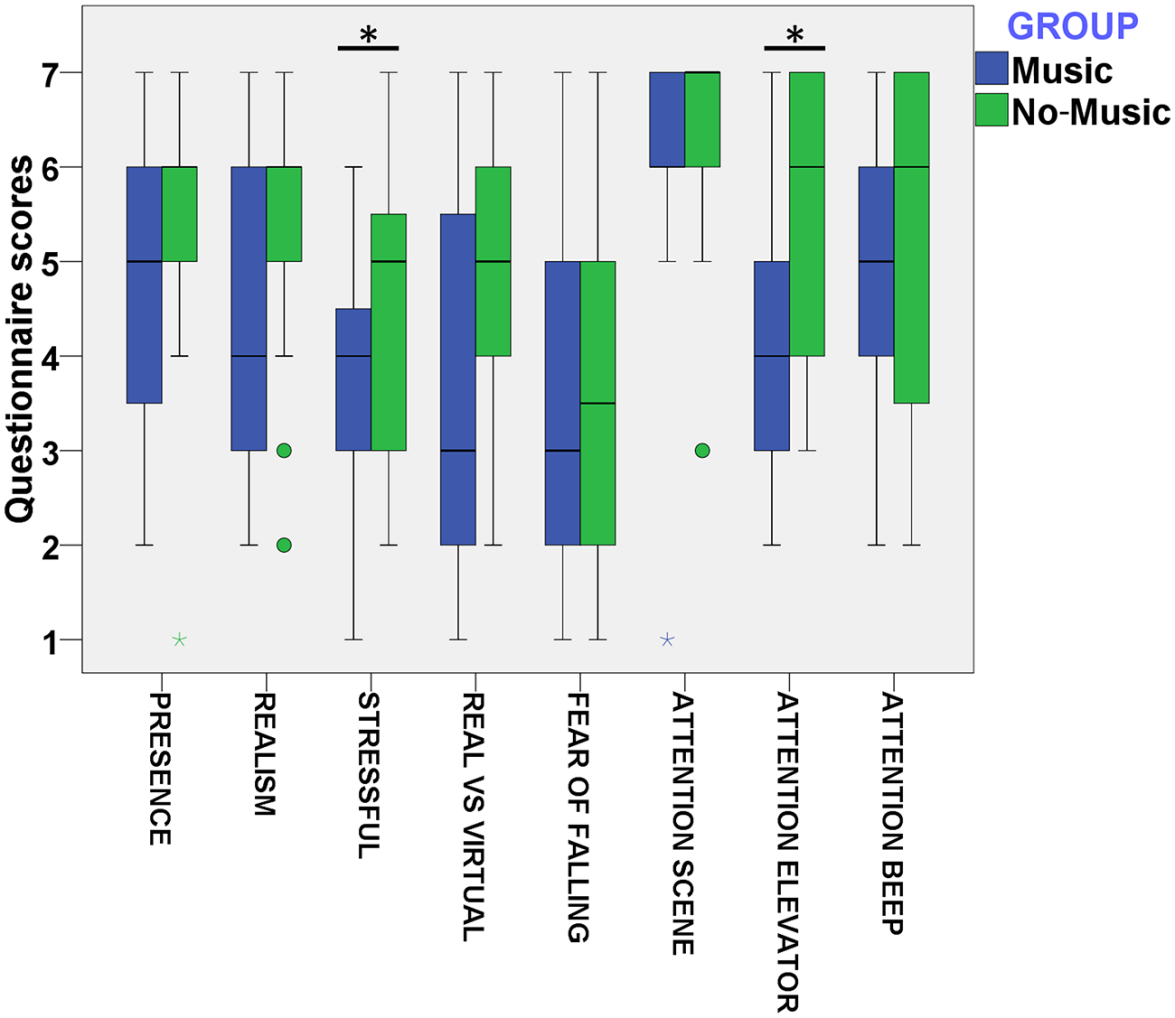
**Boxplot of VR related questions (**Table 3**) for the variables *presence*, *realism*, *stressful*, *real vs virtual*, *fear of falling*, and *attention* paid to the *scene*, sound of the *elevator* and the *beep* sound**.

**Figure 2** summarizes the boxplots for the different questions included in the virtual reality experience questionnaire (see questions in **Table 3**). In order to assess possible differences between the conditions, we ran an ordered logistic regression analysis on each questionnaire item, including Group (music and no-music) as a fixed factor and AQ as a covariate. For the questions *presence*, *realism*, *real vs. virtual*, and *attention to scene*, no main effects of Group (presence, *p* = 0.34; realism, *p* = 0.10; real vs. virtual, *p* = 0.09; and attention to scene, *p* = 0.50) or AQ (presence, *p* = 0.33; realism, *p* = 0.22; real vs. virtual, *p* = 0.75; and attention to scene, *p* = 0.29) were found.

However, we found a significant main effect of AQ (*p* < 0.01, Odd Ratio (OR) = 1.14) and of Group (*p* = 0.05, OR = 0.30) on the *stressful* questionnaire item. This result indicates that higher AQ scores are related to greater cumulative scores in the *stressful* question. Furthermore, this result also shows that subjects in the music condition have lower cumulative scores on the *stressful* question in comparison to the no-music group. We also found a significant main effect of AQ on the *fear of falling* (*p* < 0.01, OR = 2.16) and *attention to beep* (*p* = 0.02, OR = 1.04) questions. However, we did not find a significant effect of Group in these questions (fear of falling, *p* = 0.85; attention to beep, *p* = 0.19). This indicates that higher AQ scores are related to a higher degree of fear of falling and to more attention paid to the beep sound during the virtual experience. Finally, we found a significant main effect of Group, but not for AQ (*p* = 0.50), on the *attention to elevator* (*p* = 0.01, OR = 0.20) question. This shows that participants in the music condition have lower cumulative scores on the level of attention paid to the virtual elevator in comparison to the no-music condition.

### Subjective Units of Discomfort Scale (SUDS)

We hypothesized that the increase of self-reported SUDS would be significantly higher in the *no-music* condition in comparison to the *music* condition. In order to test this we ran a mixed effects model with fixed effects Group (music and no-music) and Direction (ascending or descending) over SUDS. The random effects were over the individuals. Furthermore, AQ and Floor Level were added as covariates in this analysis.

**FIGURE 3:**
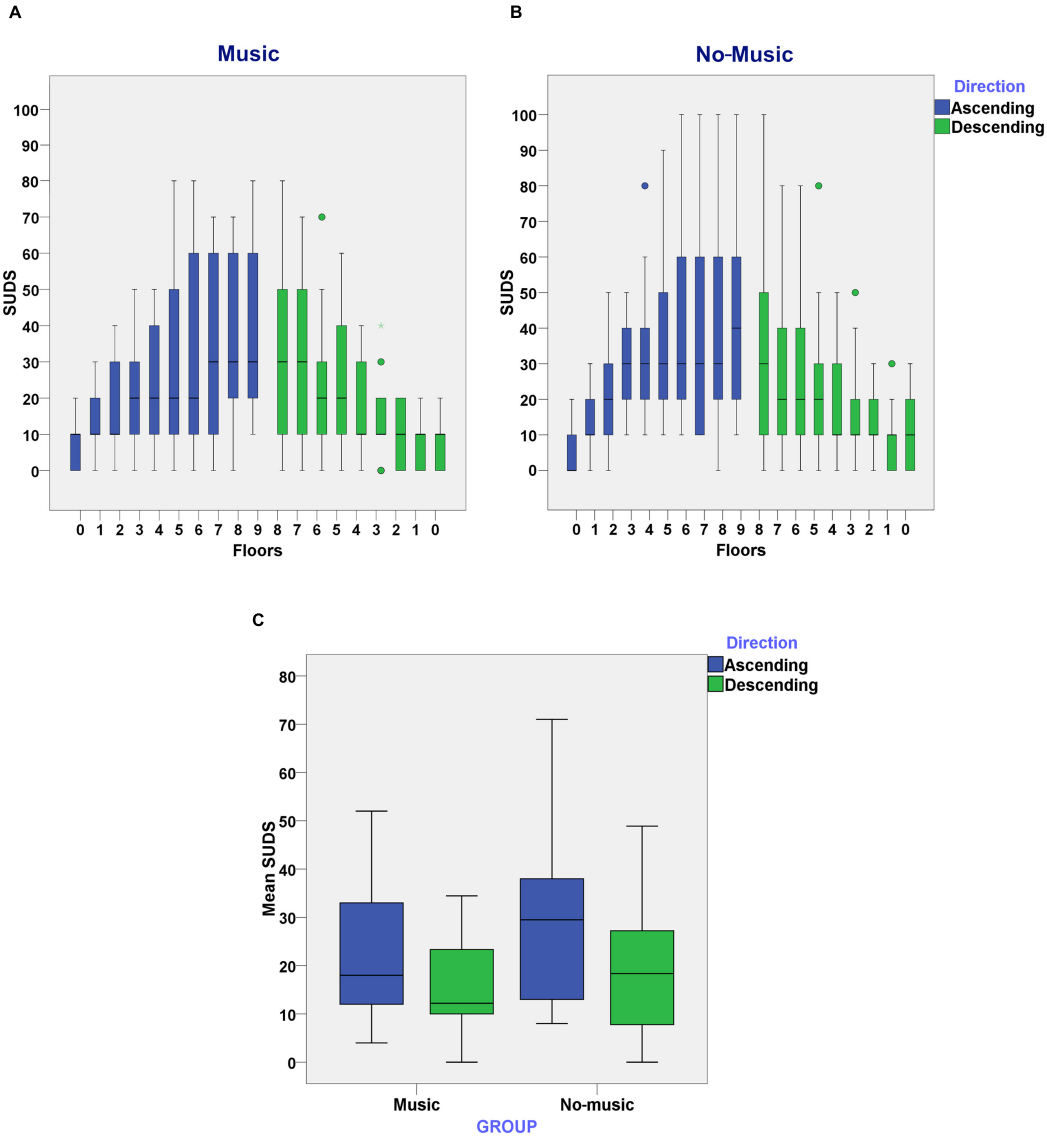
**(A)** Subjective Units of Discomfort Scores (SUDS) reported on each floor in the music condition while subjects ascended and descended in the elevator; **(B)** SUDS reported on each floor in the no-music condition while subjects ascended and descended in the elevator; **(C)** Mean of SUDS reported as subjects ascended or descended through the elevator in the music and no-music condition.

We found no significant effects for Group [*F*(1,39) = 1.16, *p* = 0.29]. However, there was a significant effect for AQ [*F*(1,39) = 18.62, *p* = 0.04], Direction [*F*(1,702) = 53.47, *p* < 0.01] and Floor Level [*F*(1,702) = 14.92, *p* < 0.01] on SUDS. This shows that a higher degree of fear of heights, shown by the AQ scores, predicted higher degrees of self-reported anxiety during the virtual experience. Furthermore, as shown in **Figure 3**, SUDS scores were significantly higher when participants ascended in the elevator in comparison to when they descended. Similarly, reported SUDS scores increased significantly when participants ascended to a higher floor.

### State-Trait Anxiety Inventory

Independent sample *t*-tests were carried out in order to assess possible differences in trait anxiety between the music and no-music conditions. No significant differences were found between the music (*Mean* = 14.58, *SD* = 6.02) and no-music (*Mean* = 16.30, *SD* = 7.32) conditions in trait anxiety. One subject belonging to the music condition was excluded only from this analysis due to missing data in this questionnaire.

**FIGURE 4:**
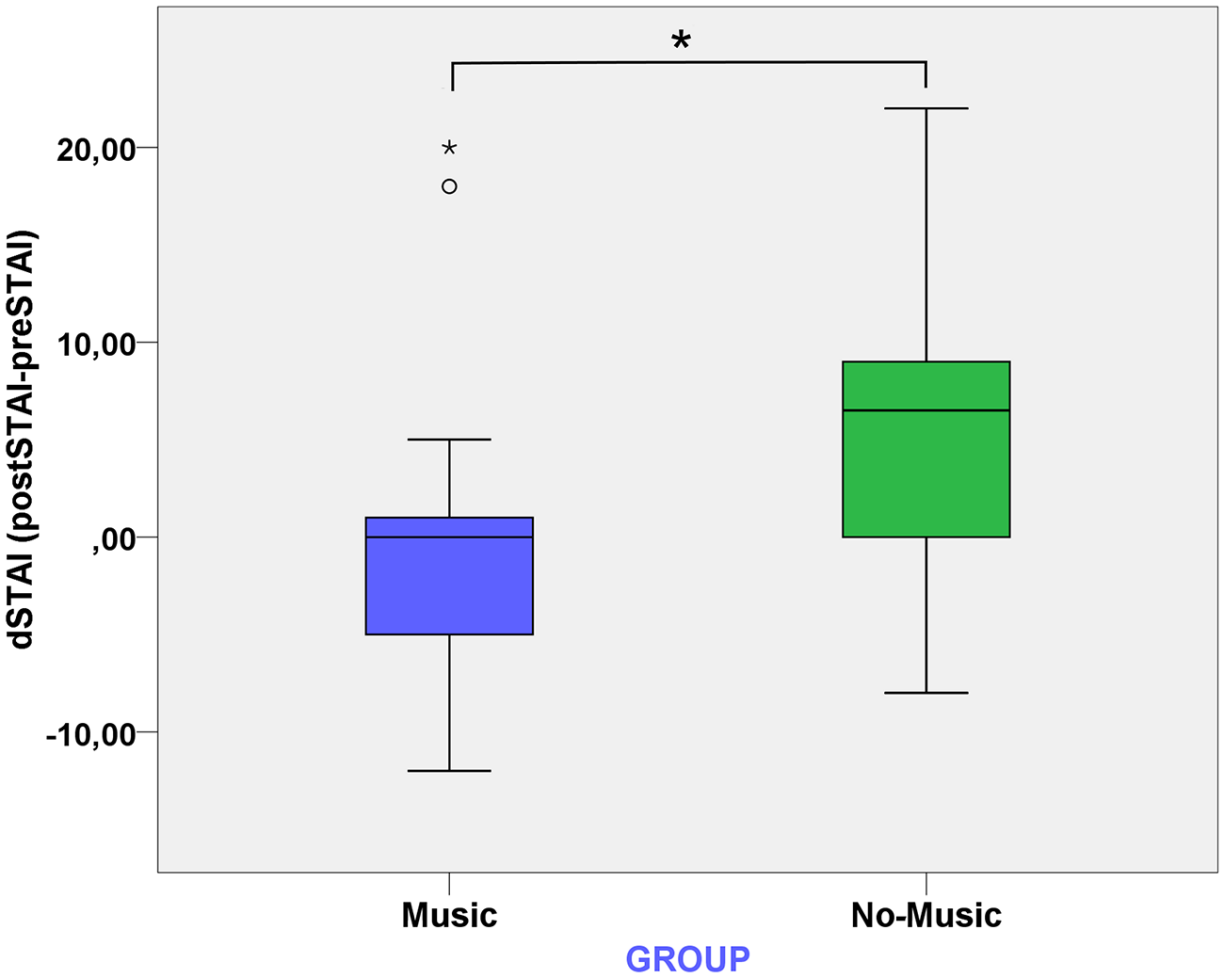
**Boxplot of dSTAI, which is the difference between the post-assessment STAI and the pre-assessment STAI, between the music and no-music conditions**.

Our prediction was that state anxiety would increase significantly in the *no-music* condition, but not in the *music* condition. In order to identify possible changes associated to the presence or absence of music during the virtual experience, the analysis was carried out using one-way ANCOVA of *dstai* (*Poststai*-*Prestai)* on the factor Group (*music* and *no-music*) and the AQ score as the covariate. As can be seen in **Figure 4**, there are two clear outliers in the music condition. When we ran the analysis including this outliers, we found a significant main effect for Group [*F*(1,35) = 4.68, *p* = 0.034, η^2^ = 0.12] and AQ [*F*(1,35) = 10.29, *p* = 0.025, η^2^ = 0.14]. However, when we ran the analysis excluding these two outliers, Group [*F*(1,33) = 12.73, *p* < 0.01, η^2^ = 0.28] remains significant with a higher effect size, while AQ [*F*(1,33) = 2.39, *p* = 0.13, η^2^ = 0.07] is not significant anymore. This result indicates that while participants in the no-music condition had a significant increase in state anxiety after the virtual experience (dSTAI *Mean* = 5.45, *SD* = 7.25), those in the music condition did not have a significant increase in their state anxiety and in some cases this anxiety was even reduced (dSTAI *Mean* = -2.31, *SD* = 4.85).

### Autonomic Perception Questionnaire

In order to identify possible changes associated to the presence or absence of music in the Autonomic Perception Questionnaire, the analysis of data was carried out using one-way ANCOVA were Group (presence or absence of music) was included as the independent variable and *dAPQ* (*PostAPQ*-*PreAPQ)* was defined as the dependent variable. In order to control for the level of fear of heights, the AQ scores were included as a covariate in this analysis. No significant effects were found for Group and AQ.

### Heart Rate (HR)

Two mixed effects models with fixed effects Group (music and no-music) were carried out in order to analyze HR responses as subjects ascended or descended through the elevator, respectively. The random effects were over the individuals. Furthermore, AQ and Floor Level were added as covariates in this analysis. We predicted that heart rate would be significantly higher in the no-music in comparison to the music condition, and also that it would be significantly influenced by the degree of fear of heights.

**FIGURE 5:**
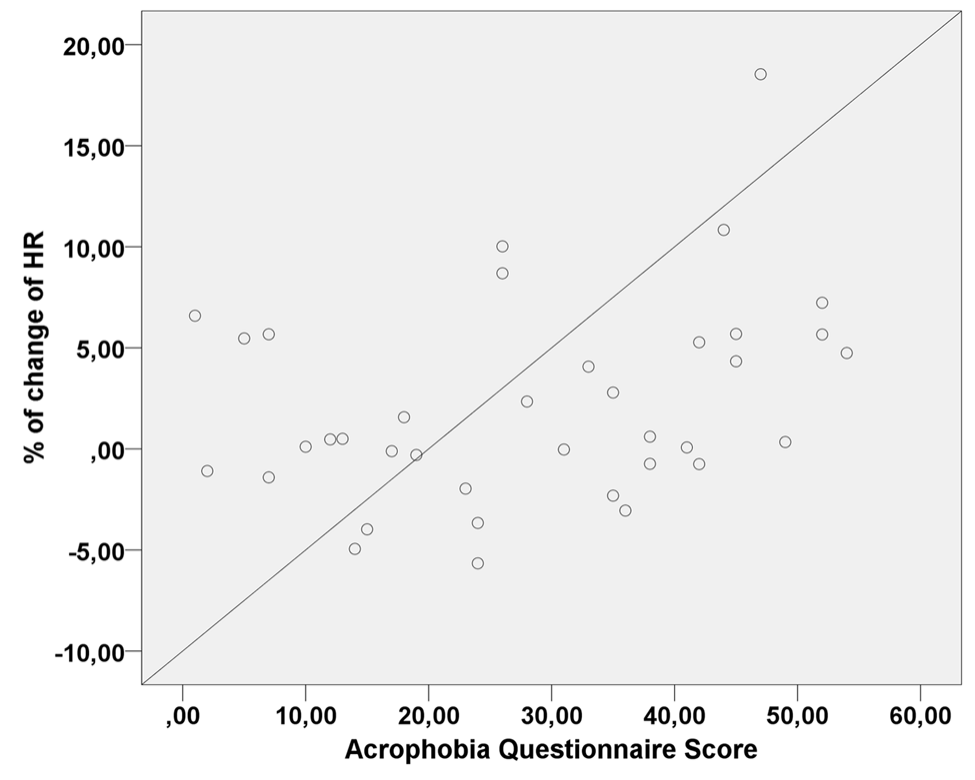
**Scatter plot of the linear correlation between Acrophobia Questionnaire (AQ) scores and percentage of change in heart rate (HR) values when subjects ascended in the elevator**.

For HR measures recorded when subjects ascended, no significant effects were found for Group [*F*(1,38) = 0.61, *p* = 0.44] and Floor Level [*F*(1,38) = 3.28, *p* = 0.07]. However, there was a significant effect of AQ [*F*(1,38) = 4.46, *p* = 0.04] on HR. This indicates that higher degrees of fear of heights are related to increase HR as subjects ascend through the virtual elevator. No significant effect for Group [*F*(1,38) = 0.13, *p* = 0.72], Floor Level [*F*(1,304) = 1.78, *p* = 0.18], or AQ [*F*(1,38) = 0.36, *p* = 0.55] were found in HR measures recorded when subjects descended. As it can be seen on **Figure 5**, AQ scores positively influenced average HR when subjects ascended in the virtual elevator.

### Skin Conductance

We hypothesized that skin conductance would be significantly higher in the no-music in comparison to the music condition. Two mixed effects models with fixed effects Group (music and no-music) were carried out in order to analyze skin conductance responses as subjects ascended or descended through the elevator, respectively. The random effects were over the individuals. Furthermore, AQ and Floor Level were added as covariates in this analysis.

For skin conductance measures recorded when subjects ascended, we found a significant effect of Floor Level [*F*(1,312) = 6.42, *p* < 0.01]. However, no significant effect were found for Group [*F*(1,39) = 0.40, *p* = 0.53] or AQ [*F*(1,39) = 0.31, *p* = 0.86]. Furthermore, no significant effect on Group [*F*(1,39) = 0.70, *p* = 0.46], Floor Level [*F*(1,39) = 0.03, *p* = 0.85], or AQ [*F*(1,39) = 0.24, *p* = 0.65], was found in skin conductance when subjects descended.

### Balance Parameters

We ran a mixed effects model with fixed effects Group (music and no music) and Direction (ascending or descending) over media-lateral and anterior – posterior sway measures. The random effects were over the individuals. Furthermore, AQ and Floor Level were added as covariates in this analysis.

No significant effect of Group [*F*(1,35) = 0.34, *p* = 0.57], Direction [*F*(1,682) = 0.02, *p* = 0.88], Floor Level [*F*(1,682) = 1.26, *p* = 0.26], and AQ [*F*(1,35) = 0.97, *p* = 0.33], were found for media-lateral sway. Concerning anterior-posterior sway, we found a significant effect for Floor Level [*F*(1,682) = 4.82, *p* = 0.03], but not for Group [*F*(1,35) = 0.003, *p* = 0.96], Direction [*F*(1,682) = 2.13, *p* = 0.14], and AQ [*F*(1,35) = 1.36, *p* = 0.25]. This means that as subject’s moved through the different floors of the building, the anterior-posterior sway increased.

## Discussion

The results obtained in this experiment indicate that music can to some extent facilitate post-stress recovery in subjects with different degrees of fear of heights. This was true in an immersive virtual reality experience that depicted a virtual platform moving up and down a building of 350 m. However, we could only observe the anxiolytic effect caused by music in subjective responses taken after the experience, and not in self-reported anxiety scores during the experience or physiological measures. These results are in line with those obtained by Burns et al. (2002) and Labbé et al. (2007) who found significant reductions in self-reported anxiety after doing a stressful arithmetic task in the presence of music.

In contrast, our results partially corroborate the results obtained by Knight and Rickard (2001), Khalfa et al. (2003), and Sandstrom and Russo (2010) since they found that music not only had an impact on a subjective level but also on cardiovascular and electrodermal responses during and after passing through a stressful situation based on preparing and giving a public talk. Our results also partially support the results of Chafin et al. (2004) and Lai et al. (2008), who found subjective and physiological reductions of anxiety while doing an exam or a complex arithmetic task in the presence of music.

A possible explanation for the diverse effects of music on modulating induced stress may be due to the control or not of the prior level of anxiety that the different types of stressors caused the experimental subjects. For example, one can employ the task of doing an exam or preparing a public talk, but these activities are not necessarily stressful to all people. In the current study we thoroughly controlled for the prior level of fear of heights that subjects experienced and analyzed to what extent the degree of fear influenced the dependent variables. The virtual reality scenario had an observable effect on all levels of responses since we observed significant differences in subjective units of discomfort, heart rate, electrodermal responses, and mean sway velocity, between the baseline (ground level) and top floor. However, a plausible explanation for the lack of results of music on SUDS and physiological responses might be that the virtual situation ended up being so stressful that the effect of music was not sufficient to affect the participants during the experience, but only afterward when recovering from stress. One could argue that experiencing oneself over a platform at 350 m gives rise to a deeply rooted life-threatening fear. The fact that the event is virtual does not eliminate anxiety when faced with a stressful situation, as long as subjects feel present in the virtual environments, which has been consistently demonstrated (for a review see Sanchez-Vives and Slater, 2005).

One of the most studied topics on music and mood regulation is the role that musical preference and genre play. Consistent with previous studies, in this experiment we have shown that familiarity or selection of the musical piece is not completely necessary to observe the anxiety reducing effect of music (Thayer and Levenson, 1983; Knight and Rickard, 2001; Burns et al., 2002; Chafin et al., 2004; Labbé et al., 2007; Yamamoto et al., 2007; Lai et al., 2008; Sandstrom and Russo, 2010). Using the favorite music of participants has the inherent bias of making subjects unconsciously overestimate the effects of music (Elliott et al., 2011). The music used in this study did not have any previous emotional meaning for subjects since it was a newly computer-generated instrumental piece, and the composition parameters were based on prior research (Pelletier, 2004; Zentner et al., 2008). Further research should consider which types of music seem to be more effective for anxiety reduction.

These results are relevant for future clinical applications that use music as means to reduce anxiety in real contexts. Nilsson (2008) concludes in his review that music can greatly reduce pre-operative anxiety. In this sense, we think that the use of music in a hospital setting is appropriate since it should help patients to reduce their anxiety, experience less pain and make hospital stays less traumatic. In the same manner, different situations that induce anxiety in large populations like exams, dental consultations and, working contexts, might benefit from the presence of music.

We have found that there is a lack of research concerning the role of music in psychological treatments; therefore its use in this context should be with caution, although, studies that have focused on the use of music in phobias and cognitive restructuring have found a positive relation between music and treatment outcomes (Eifert et al., 1988; Kerr et al., 2001). In the field of music therapy, there have been many studies that point out the possible benefits of the use of music in psychological disorders. Although, meta-analytic reviews have highlighted that the methodology used in this studies should be stricter and that more research is needed to have conclusive results.

In this experiment, we have induced fear of heights in some subjects and results show that post-experience subjective anxiety is lower in the condition that included music compared to no music. This suggests that music might be used in some phases of an exposure therapy, for example, in the first phases of the treatment music might reduce dropouts, facilitate the acceptance of exposure to the feared stimuli and induce positive emotions. However, caution should be taken since gradually the patient should learn to cope with the situation without the presence of music, so music does not become an avoidance coping strategy. There is a need of more research focus on the impact of music on clinical populations (Koelsch, 2014). A possible explanation of the effect of music in this context is a evaluative conditioning effect were an association is created between an aversive (heights) and positive (music) stimuli.

Another contribution of this study was to the field of presence in virtual environments, presenting additional evidence of the effectiveness of virtual reality for inducing anxiety responses in relation to the prior level of fear participants had for a specific situation. We found that higher levels of fear of heights in the real world were related to higher scores in state anxiety, SUDS, and HR. These results support the substantial number of studies that have shown that virtual reality exposure therapy is a viable treatment option for anxiety disorders (Powers and Emmelkamp, 2008). Based on these results we conclude that virtual reality is a useful tool for studying different psychological phenomena while preserving ecological and internal validity, and eventually also as tool for therapy.

Our results are compatible with different mechanisms of action of music inducing a reduction of anxiety: either acting directly as a distractor or directly influencing mood. Future studies should address this question by comparing the use of music against other distracting stimuli (e.g., hearing other type of sound, like nature sounds, people speaking, etc.). Another limitation is that we did not screen participants for possible comorbid psychopathology and groups were not balanced by gender. Future studies should control for possible comorbid psychopathology with the use of a standardized questionnaires, as well as control for and analyze the influence that gender can play on anxiety responses and the possibly differing effects of music on people of different genders. Finally, our 30s physiological measures (SCL, HR) were enough to detect changes at different heights, but did not allow assessing a possible enhancement of parasympathetic activity through music. Future studies should consider recording physiological signals, such as electrocardiography (ECG) for longer time periods in order to analyze responses such as the Respiratory Sinus Arrhythmia (RSA) which has been linked to parasympathetic activity.

## Author Contributions

SS, MVSV, FV, and MS conceived of the project; SS with the help of MVSV, MS, IB, AP, and JP designed the experiments; AP, IB, and JP programmed the experimental scenario; SS performed the experiments with the help of IB; SS with the help of MS and IB analyzed the data; SS wrote the paper with the help of all authors; all authors read and approved the manuscript.

## Conflict of Interest Statement

The authors declare that the research was conducted in the absence of any commercial or financial relationships that could be construed as a potential conflict of interest.
